# *Agaricus bisporus* stipe fed to dairy heifers: effects on growth performance, immunity and antioxidant capacity, and rumen microbiota

**DOI:** 10.3389/fvets.2025.1556330

**Published:** 2025-03-21

**Authors:** Peng Jia, Chunguang Lu, Xingyu Yang, Xueyuan Jiang, Hulong Lei, Naisheng Lu, Dong Xia

**Affiliations:** ^1^Key Laboratory of Livestock and Poultry Resources (Pig) Evaluation and Utilization of Ministry of Agriculture and Rural Affairs, Shanghai Engineering Research Center of Breeding Pig, Institute of Animal Husbandry and Veterinary Science, Shanghai Academy of Agricultural Sciences, Shanghai, China; ^2^Jinshan District Agricultural Technology Extension Center, Shanghai, China; ^3^Jinshan Distict Animal Disease Control Center, Shanghai, China

**Keywords:** heifers, mushrooms, *Agaricus bisporus*, growth performance, serum immunity, serum antioxidant, rumen microbiota

## Abstract

**Introduction:**

Mushrooms are rich in protein, polysaccharides, and other nutrients as a natural homology of medicine and food species, among which *Agaricus bisporus* is one of the most widely cultivated and consumed mushrooms worldwide. The mushroom stipe is a by-product of the mushroom industry that can be utilized as a feed resource based on its rich nutritional value. This study examined the effects of *Agaricus bisporus* stipe (ABS) as a feed additive on growth performance, blood indexes, rumen fermentation parameters, nutrient digestion and utilization, and rumen microbiota of heifers.

**Methods:**

Twenty Holstein heifers (268 ± 9.43 d of age) were blocked in 10 groups of 2 heifers according to their live weights and ages, and within each group, the 2 heifers were randomly allocated to 1 of 2 treatments: control diet (CON), control diet plus ABS (ABS, 150 g·DM/animal/d).

**Results:**

Heifers supplemented with ABS had higher average daily gain and a tendency to decrease feed conversion ratio, but rumen fermentation parameters were similar between the two treatments. Heifers in ABS had increased IgG, IgA, IgM, SOD, T-AOC, and GSH-Px concentrations while decreasing MDA concentration than heifers in CON. Although energy and nitrogen utilization were similar between treatments, the apparent total-tract digestibilities of NDF and ADF were higher for ABS than for CON heifers. Feeding ABS increased the relative abundance of one phylum (Verrucomicrobiota), two genera (*Akkermansia* and *Ligilactobacillus*), and two species (*Akkermansia muciniphila* and *Ligilactobacillus murinus*) in the rumen of heifers.

**Discussion:**

In conclusion, heifers receiving ABS have greater growth performance, improved serum immune and antioxidant indexes, increased the relative abundance of some rumen bacteria, and higher nutrient digestion than CON heifers.

## 1 Introduction

Mushrooms are an important source of nutrients worldwide, rich in protein and dietary fiber but low in fat (1). They are an excellent source of several micronutrients (including vitamin B_3_, vitamin B_5_, copper, selenium, and phosphorus), and it is important to note that mushrooms are the only vegetarian source of vitamin B_12_ and vitamin D (2). In addition, mushrooms are a potential source of bioactive molecules that promote health, including glycoproteins, triterpenes, chitin, *α* and *β*-glucan, mannan, xylans, and galactans (3). Therefore, mushrooms are natural homologies of medicine and food species, with anti-oxidative, immunity-boosting, anti-viral, anti-carcinogenic, and anti-tumor effects (4). According to the Food and Agriculture Organization (5) of the United Nations, global mushroom production increased fivefold from 2000 to 2023, reaching 44 million tons based on the nutritional value of mushrooms. The global market sales of mushrooms reached $399.67 billion in 2023, with China being the largest trading market, followed by Europe and North America, and it is predicted that the global mushroom market size will gradually expand.^1^

*Agaricus bisporus* is a member of the Agaricaceae family (6), also known as the white button mushroom or metacercaria (7), and is one of the most widely consumed and cultivated mushrooms worldwide (8). *Agaricus bisporus* is rich in nutrients, including essential amino acids, dietary fiber, B-vitamins, and vitamin D_2_ (9). *Agaricus bisporus* is also used as an effective alternative to animal protein due to their crude protein (CP) content of 19–38% on a dry matter (DM) basis (10). The main active components of the *Agaricus bisporus* are polysaccharides, which mainly include *β*-glucan polymers, xylans, galactans, mannans, and chitin (11, 12). Extensive studies have shown that the polysaccharide has antioxidant, antibacterial, immunomodulatory, treatment of diabetes and cardiovascular disease, prevention and treatment of various cancers, regulation of intestinal microbiota diversity and abundance, and other biological activities (11, 13). In addition, *Agaricus bisporus* is a good source of selenium (2.82 ± 1.48 mg/kg DM), which is widely recognized to reduce the risk of many health problems in humans and animals (14). In comparison to *Pleurotus eryngii* (DC.) Quel, *Grifola frondosa* (Dicks.) Gray, *Pleurotus ostreatus*, and *Lentinus edodes*, *Agaricus bisporus* exhibits greater antioxidant activity, attributed to its natural antioxidants, including phenols, serotonin, and tocopherols (6).

The *Agaricus bisporus* stipe (ABS), which measures about 3 cm at the root, is frequently removed during the *Agaricus bisporus* production process to ensure the cleanliness and quality of *Agaricus bisporus* products. *Agaricus bisporus* has the characteristics of a low planting environmental footprint, but by-products account for 20% of the total weight of *Agaricus bisporus* (15), which affects the ecological sustainable development of the mushroom industry. Notably, ABS has chitin comparable to mushroom caps and a high protein content, making it a valuable by-product. Therefore, ABS can be applied to animal feed, and some literature has reviewed the reuse of nutrients and bioactive ingredients in mushrooms (15–17). The replacement of 10 and 20% conventional feedstuffs with *Agaricus bisporus* did not adversely affect the growth performance of lambs (18), while the addition of 20 g/kg ABS in the basal diet improved the feed conversion ratio of broilers (19). Furthermore, adding *Agaricus bisporus* to the diet increased the meat color index of mutton (20), and increased the added value of quail eggs such as reducing yolk 1,1-diphenyl-2-picrylhydrazyl and malondialdehyde (MDA) content (21). Intestinal health is essential for both dietary nutrient absorption and animal immunity. In some previous studies, mushrooms have been demonstrated to improve intestinal morphology in broilers by increasing the ratio of villus height to crypt depth in the jejunal and villus height in the ileum (19), as well as by reducing the number of *Escherichiacoli* and *Enterobacteriaceae* in the rectum to reduce the average diarrhea severity in broilers (22). It is unclear whether ABS affects the rumen microbial community, in particular, the application effect of ABS in cattle diet. The objective of the study was to evaluate the effects of ABS supplementation on the growth performance and rumen bacterial populations of dairy heifers in a commercial herd.

## 2 Materials and methods

The experiment was performed from June 8, 2024 to August 28, 2024 at the Yinan Dairy Farm in Shanghai, China. Experimental animal handling procedures were approved by the Animal Care and Use Committee of the Shanghai Academy of Agricultural Sciences.

### 2.1 Heifers and experimental design

A total of 20 Holstein heifers (mean ± SD; 268 ± 9.43 d of age, 335.8 ± 31.71 kg of BW) were used in a randomized complete block design and were blocked in 10 groups of 2 cows according to initial age and BW. Heifers from each block were randomly assigned into 1 of 2 treatments. The treatments were as follows: basal diet without ABS (control, CON) and diet with fresh ABS (ABS). Heifers were fed diets as total mixed ration (TMR) twice daily at 04:00 and 11:00 h, and the TMR offered was adjusted daily to allow a 5–10% refusal rate. The dosage of ABS was 150 g/animal per day based on DM basis (19), and the nutrient composition of ABS is shown in Table 1. The ABS was fed to each heifer before feeding TMR to ensure that each heifer received a set amount of ABS. The ingredient and nutrient composition of the basal diet are shown in Table 1. The TMR was formulated to contain 50.77% neutral detergent fiber (NDF), 33.55% acid detergent fiber (ADF), 17.26% CP, and minerals and vitamins to meet NRC (2001) (23) requirements to maintain a growth rate of ~0.9 kg/d for heifers. Experimental diets contained (on a DM basis) 25.8% corn silage, 21% soybean meal, 15% ground corn, 9.8% oatgrass hay, 8.6% soybean hulls, 6.1% alfalfa hay, 6.1% wheat straw, 6.1% dried distillers grains with solubles, and 1.5% mineral mix. Control and experimental heifers were fed individually in a tiestall barn, with an average feed bunk spacing of 0.80 m per heifer. During the experiment, each heifer was allowed to drink freely from an individual water bowl throughout the day. The experimental period lasted 50 d, with the first 10 d used for diet adaptation and the last 40 d for data and sample collection.

**Table 1:** Ingredient and chemical composition (percentage of DM) of the diets and ABS.

Item	Compositions (% of DM or as indicated)
TMR	
Ingredients	
Corn silage	25.8
Soybean meal	21.0
Ground corn	15.0
Oatgrass hay	9.8
Soybean hulls	8.6
Alfalfa hay	6.1
Wheat straw	6.1
Distillers dried grains with solubles	6.1
Mineral and vitamin premix^1^	1.5
Chemical composition^2^	
DM	69.49
OM	97.13
NDF	50.77
ADF	33.55
CP	17.26
EE	3.09
Ca	0.80
P	0.41
ME, Mcal/kg	2.5
ABS	
DM	7.53
OM	99.78
NDF	29.69
ADF	12.82
CP	30.12
EE	1.94
GE, Mcal/kg	19.15
Ca	0.03
P	0.13

1 The premix contained the following components per kg: Cu, 350 mg; Fe, 150 mg; Zn, 1,375 mg; Mn, 1,000 mg; I, 15 mg; Se, 18 mg; Co, 6 mg; vitamin A, 888,890 IU; vitamin D, 111,111 IU; vitamin E, 8,333 mg.

2 DM, dry matter; BW, body weight; OM, organic matter; NDF, neutral detergent fiber; ADF, acid detergent fiber; EE, ether extract.

### 2.2 Data and sample collection

#### 2.2.1 Diet and feed ingredients

The amount of feed delivered and refused for each heifer was weighed and recorded daily. The TMR and ABS were sampled daily, mixed on a weekly basis, subsampled, and then dried at 55°C under forced air for 48 h to evaluate DM concentrations. The dietary samples were then ground through a mill fitted with a 1-mm screen and stored for subsequent nutritional analysis. Feed samples were tested for DM (method 930.15, AOAC International, 2000), CP (method 990.03; AOAC International, 2000), ash (method 942.05, AOAC International, 2000), ether extract (EE; method 920.29 AOAC International, 2000), and minerals (method 985.01, AOAC International, 2000). Moreover, according to the method of Van Soest et al. (24), samples were treated with heat-stable *α*-amylase and sodium sulfite, and NDF and ADF were analyzed sequentially, which was applied to the Ankom^200^ fiber analyzer (Ankom Technology, Macedon, NY, United States). The gross energy (GE) content in feed samples was also analyzed by a bomb calorimeter (Calorimeter Parr6400, Parr Instrument Company, Moline, IL, USA). Nutrient compositions of the TMR and ABS are shown in Table 1, respectively. Daily refusals were converted to a DM basis and then subtracted from the daily dietary DM provided to determine the daily DMI for each heifer.

#### 2.2.2 Nutrient digestibility, energy and nitrogen utilization

From d 46 to 49, 4 heifers in each group were randomly selected for measurement of apparent total-tract nutrient digestibility, energy utilization, and nitrogen utilization. Feed consumption was obtained on a daily basis by weighing feed delivered to and refused by heifers. Diets and refusals were sampled daily, dried for 48 h at 55°C, ground through a mill fitted with a 1-mm screen, compounded over 4 days, and then sealed and stored for subsequent nutrient (DM, OM, CP, EE, NDF, ADF, and GE) analysis. Total fecal and urine output were collected and weighed from each heifer for 4 consecutive days. Representative (2%, on a fresh matter basis) samples of thoroughly mixed feces and urine were collected daily. Fecal samples were stored at −20°C until nutritional analysis was performed. The urine sample was diluted with 4 volumes of 0.072 mol/L sulfuric acid and stored at −20°C for further analyses. Fecal samples were dried for 72 h at 55°C and then ground using a mill to pass a 1-mm sieve. Fecal samples were composited in proportion to the DM for each heifer based on the daily fecal outputs. Ground fecal samples were analyzed for DM, OM, CP, EE, NDF, ADF, and GE using the same methods as diet samples. Measurement of urine CP and GE was performed as described in Jia et al. (25). Apparent total-tract nutrient digestibility was calculated according to the following equation: nutrient digestibility = 100% − [(fecal output, kg) × (fecal nutrient content, %)/(DMI, kg) × (feed nutrient content, %)]% (26).

#### 2.2.3 BW and blood measurements

At the initiation and termination of the experiment, each heifer was weighed before morning feeding on 2 consecutive days using an electronic scale (Shanghai Yaohua Weighing System Co., Ltd., Shanghai, China). On the last day of the experiment, blood samples were collected from the tail vein of each heifer into serum separator tubes (BD Vacutainer, Franklin Lakes, NJ, United States) before morning feeding. After collection, the samples were immediately stored in the shade and centrifuged at 3500 r/s for 20 min. The supernatant was pipetted into two 2-mL tubes and stored at −20°C for detection. Blood serums were evaluated for immunoglobulin A (IgA), immunoglobulin G (IgG), immunoglobulin M (IgM), superoxide dismutase (SOD), glutathione peroxidase (GSH-Px), MDA, total antioxidant capacity (TAC) by Beijing Jinhaikeyu Biotechnology Development Co., Ltd. (Beijing, China).

#### 2.2.4 Rumen measurements

On the last day of the experiment, 6 heifers from each group were randomly selected to collect rumen fluid to obtain a representative sample. At 2 h after morning feeding, rumen fluid from heifers was collected using an oral stomach tube. The first 200 mL of fluid was discarded to avoid contamination of the sample by saliva, and 100 mL of rumen fluid was collected. Rumen fluid samples were measured for pH value (basic pH meter PB-20, Startorius AG, Germany) immediately after collection. Subsequently, 2 2-mL rumen fluid samples were collected and frozen in liquid nitrogen, and stored at −80°C until DNA extraction. The remaining rumen fluid was filtered through 4 layers of cheesecloth, transferred to 2 10-mL tubes with either 200 μL of 50% sulfuric acid or 2 mL of 25% meta-phosphoric acid, and stored at −20°C for subsequent analysis of ammonia nitrogen (NH_3_-N) and volatile fatty acids (VFA), respectively. The colorimetric assay described by Chaney and Marbach (27) was used to determine rumen NH_3_-N using a microplate spectrophotometer (Spectra max 190, Molecular Devices, United States). Sample preparation and measurement of VFA were performed according to the protocol described in Qumar et al. (28), using a gas chromatography apparatus (Agilent 6,890 N; Agilent Technologies Canada Inc., Mississauga, ON, Canada) equipped with a capillary column of 30-m length, 0.53-mm diameter, and 0.53-μm film thickness (Trace TR Wax, Thermo Fisher Scientific, Waltham, MA, United States).

#### 2.2.5 DNA extraction and PCR amplification

Genomic DNA was extracted from rumen fluid samples using a commercial kit (TIANamp Stool DNA Kit DP328, Tiangen Biotech, Beijing, China) in accordance with the manufacturer’s protocols. The concentration and purity of the extracted DNA were determined by 1.0% agarose gel electrophoresis and a NanoDrop 2000 spectrophotometer (Thermo Fisher Scientific, Waltham, MA, United States). A pair of primers, 338F (5′-ACTCCTACGGGAGGCA GCAG-3′) and 806R (5′-GGACTACHVGGGTWTCTAAT-3′) was used to amplify 16S rRNA genes. PCR amplification was conducted under the following conditions: 95°C for 2 min, followed by 27 cycles at 95°C for 30 s, 55°C for 30 s, and 72°C for 60 s and a final extension at 72°C for 5 min. PCR reactions were carried out in triplicate within a 20 μL mixture comprising 10 μL of 2X Pro Taq, 0.8 μL of each primer (5 μM), 10 ng of template DNA, and double-distilled H_2_O. The products OF the PCR amplification were extracted from 2% agarose gels and purified using a commercial kit (AxyPrep DNA Gel Extraction Kit, Axygen Biosciences, Union City, CA, United States) according to the manufacturer’s instructions. Purified amplicons were pooled in equimolar amounts, and sequencing of the amplicons was performed by Shanghai Biozeron Biotechnology Co., Ltd. (Shanghai, China).

#### 2.2.6 Sequence processing and analysis

The amplicon sequences were filtered using FASTP (v0.19.6) (41) and subsequently merged with FLASH (v1.2.11) (29). The reads were processed on the QIIME2 platform (v2022.2) (30), where high-quality sequences were denoised with the DADA2 plugin. This process completed sequence quality control and construction of amplicon sequence variants (ASV) (31). Using a pretrained QIIME2-compatible SILVA 138 database (32) for bacteria, ASV was classified by a pre-fitted classifier based on scikit-learn (33) with a similarity threshold of 99%. Rarefaction analysis, utilizing ASV information based on Mothur v.1.30.1 (34), further revealed the *α*-diversity indexs, including Chao richness, abundance-based coverage estimators (ACE) index, Shannon index, and Simpson index. Beta diversity analysis was conducted using the Vegan v2.5–3 package in R studio (ver. 2023.06.2 + 561, PBC, Boston, MA, United States), where the similarity between microbial communities in different samples was assessed through principal coordinate analysis (PCoA) based on Bray-Curtis distance matrices. Spearman correlation was used to evaluate the correlations between the top 20 bacterial genera and physiological parameters in dairy heifers.

### 2.3 Statistical analyses

Data were analyzed using the SAS 9.4 (SAS Institute Inc., Cary, NC). Before analysis, data were tested for normality using the UNIVARIATE procedure, and the PROC Univariate was used to remove outliers from the statistical analysis when the absolute studentized residual values were > 3.0. The DMI, BW, apparent total-tract nutrient digestibility, energy utilization, nitrogen utilization, blood indexes, and rumen fermentation parameters were analyzed using the one-way ANOVA procedure according to the following model:


$$Y_{i}=\mu +T_{i}+e_{i}$$

where *Y*_i_ is the dependent variable, *μ* is the overall mean, *T*_i_ is the effect of treatment (i = 1, 2), and *e*_i_ is the residual error. Compared diversity and relative abundance of bacteria between the CON and ABS using the Wilcoxon rank-sum test in SPSS 19.0. Statistical significance was declared at *p* ≤ 0.05, and tendencies at 0.05 < *p* ≤ 0.10.

## 3 Results

### 3.1 Growth performance and ruminal fermentation parameters

Growth performance and ruminal fermentation parameters of heifers are presented in Table 2. Initial BW was similar between treatments (*p* > 0.05). Addition of ABS did not affect final weight but increased average daily gain (ADG) by 0.16 kg/d (*p* ≤ 0.05). The DMI did not differ (*p* > 0.05) between heifers fed control and ABS diets. However, heifers on ABS treatment had a tendency to decrease the feed conversion ratio by 9.7% (0.05 < *p* ≤ 0.10). Additionally, ABS had no effect on pH value, as well as concentrations of total VFA, and molar proportions of acetate, propionate, butyrate, isobutyrate, valerate, and isovalerate (*p* > 0.05).

**Table 2:** Effects of feeding ABS for dairy heifers on the growth performance and ruminal fermentation parameters.

Item^2^	Treatment^1^		SEM	*p*-value
	CON	ABS		
Initial BW, kg	338.8	336.1	6.916	0.854
Final BW, kg	367.9	369.3	6.683	0.922
ADG, kg/d	1.17	1.33	0.069	0.032
DMI, kg/d	6.82	6.96	0.062	0.266
Feed conversion ratio, kg of DMI/kg of ADG	5.89	5.32	0.167	0.085
NH_3_-N, mg/L	16.23	16.56	1.763	0.698
pH value	6.88	6.84	0.034	0.576
Total VFA, mM	112.62	112.12	4.798	0.962
Acetate, %	56.14	54.99	0.585	0.352
Propionate, %	26.17	25.93	0.496	0.824
Butyrate, %	16.30	17.27	0.364	0.200
Isobutyrate, %	1.07	1.03	0.047	0.703
Valerate, %	1.42	1.46	0.040	0.584
Isovalerate, %	1.51	01.50	0.046	0.861
Acetate/propionate	2.40	2.33	6.416	0.603

1 CON, basal diet; ABS, basal diet including 150 g·DM/heifer daily of fresh ABS.

2 BW, body weight; ADG, average daily gain; DMI, dry matter intake; NH_3_-N, ammonia nitrogen; VFA, volatile fatty acids.

### 3.2 Serum parameters

The influences of ABS supplementation on serum immunity and antioxidant status of heifers are shown in Table 3. Supplementation with ABS increased (*p* ≤ 0.05) the concentrations of IgG, IgA, and IgM compared to CON (11.12 vs. 10.07 mg/mL, 0.833 vs. 0.683 mg/mL, 2.921 vs. 2.480 mg/mL). Similarly, SOD, TAC, and GSH-Px increased (*p* ≤ 0.05) by 8.8, 1.55, and 80.6 U/mL for the ABS when compared with CON, respectively (111.8 vs. 103.0 U/mL, 11.21 vs. 9.66 U/mL, 1,015 vs. 934.4 U/mL). However, ABS supplementation decreased (*p* ≤ 0.05; 3.888 vs. 4.672 nmol/mL) MDA concentration compared with CON.

**Table 3:** Effects of feeding ABS for dairy heifers on serum immunity and antioxidant status.

Item^2^	Treatment^1^		SEM	*p*-value
	CON	ABS		
Serum immunity (g/L)				
IgA	0.68	0.83	0.028	0.004
IgG	10.07	11.12	0.196	0.004
IgM	2.48	2.92	0.083	0.004
Serum antioxidants (U/mL)				
TAC	9.66	11.21	0.294	0.004
GSH-Px	934.4	1,015	14.430	0.002
SOD	103.0	111.8	1.634	0.003
MDA, nmol/mL	4.67	3.89	0.147	0.004

1 CON, basal diet; ABS, basal diet including 150 g·DM/heifer daily of fresh ABS.

2 IgA, immunoglobulin A; IgG, immunoglobulin G; IgM, immunoglobulin M; TAC, total antioxidant capacity; GSH-Px, glutathione peroxidase; SOD, superoxide dismutase; MDA, malondialdehyde.

### 3.3 Nutrients digestibility, energy and nitrogen utilization

The effects of ABS on the nutrient apparent digestibility of dairy heifers are presented in Table 4. Intakes of DM, OM, NDF, ADF, and EE were not different between the CON and ABS groups (*p* > 0.05). Supplementation of ABS significantly increased the apparent total-tract digestibilities of NDF (64.81% vs. 69.46%) and ADF (64.11% vs. 68.67%) compared with the control group (*p* ≤ 0.05), but did not affect the apparent total-tract digestibilities of DM, OM, and EE (*p* > 0.05). Energy intake, output, and utilization did not differ between the two groups (*p* > 0.05), nor did nitrogen intake, output, and utilization (*p* > 0.05) (Table 5).

**Table 4:** Effects of feeding ABS for dairy heifers on the apparent total-tract digestibility of nutrients.

Item^2^	Treatment^1^		SEM	*p*-value
	CON	ABS		
Intake (kg/d)				
DM	8.53	8.52	0.061	0.825
OM	8.28	8.29	0.041	0.853
NDF	4.28	4.26	0.347	0.756
ADF	2.83	2.81	0.457	0.767
EE	0.26	0.26	0.019	0.866
Digestibility (%)				
DM	67.79	68.44	0.786	0.524
OM	71.10	72.50	0.813	0.338
NDF	64.81	69.46	1.441	0.028
ADF	64.11	68.67	1.450	0.029
EE	66.57	64.92	1.583	0.894

1 CON, basal diet; ABS, basal diet including 150 g·DM/heifer daily of fresh ABS.

2 DM, dry matter; OM, organic matter; NDF, neutral detergent fiber; ADF, acid detergent fiber; EE, ether extract.

**Table 5:** Effects of feeding ABS for dairy heifers on energy and nitrogen utilization.

Item^2^	Treatment^1^		SEM	*p*-value
	CON	ABS		
Energy intake and output (MJ/d)				
GE	156.94	154.05	0.002	0.912
Fecal E	46.91	44.94	1.183	0.315
Urinary E	5.31	4.77	0.314	0.433
Manure E	52.22	49.71	1.448	0.321
DE	110.03	109.11	1.182	0.317
ME	104.72	104.34	1.447	0.322
Energy use (%)				
Fecal E/GE	29.89	29.17	0.008	0.315
Urinary E/GE	3.38	3.09	0.002	0.433
Manure E/GE	33.27	32.27	0.008	0.321
DE/GE	70.11	70.83	0.008	0.315
ME/GE	66.73	67.73	0.009	0.321
ME/DE	94.97	95.63	0.003	0.399
Nitrogen intake and output (g/d)				
Intake N, g/d	237.34	240.43	0.584	0.893
Fecal N g/d	68.10	65.63	1.864	0.550
Urinary N	65.24	61.95	2.090	0.474
Manure N	133.33	127.58	2.696	0.321
DN	169.24	174.80	2.090	0.204
Retained N	104.00	112.85	2.981	0.148
Nitrogen use (%)				
Fecal N/Intake N	28.69	27.30	0.805	0.429
Urinary N/Intake N	27.50	25.77	0.900	0.378
Manure N/Intake N	56.18	53.07	1.193	0.213
DN/Intake N	71.31	72.71	0.805	0.429
Retained N/Intake N	43.82	46.94	1.193	0.213
Retained N/DN	61.42	64.56	1.307	0.259

1 CON, basal diet; ABS, basal diet including 150 g·DM/heifer daily of fresh ABS.

2 GE, gross energy; E, energy; Manure E, Fecal E + Urinary E; DE, digestible energy: GE − Fecal E; ME, metabolizable energy, DE − Urinary E. N, nitrogen; Manure N, Fecal N + Urinary N; DN, digestible nitrogen, Intake N − Fecal N; Retained N, DN − Urinary N.

### 3.4 Diversity and relative abundance of the rumen bacteria

**Figure 1:**
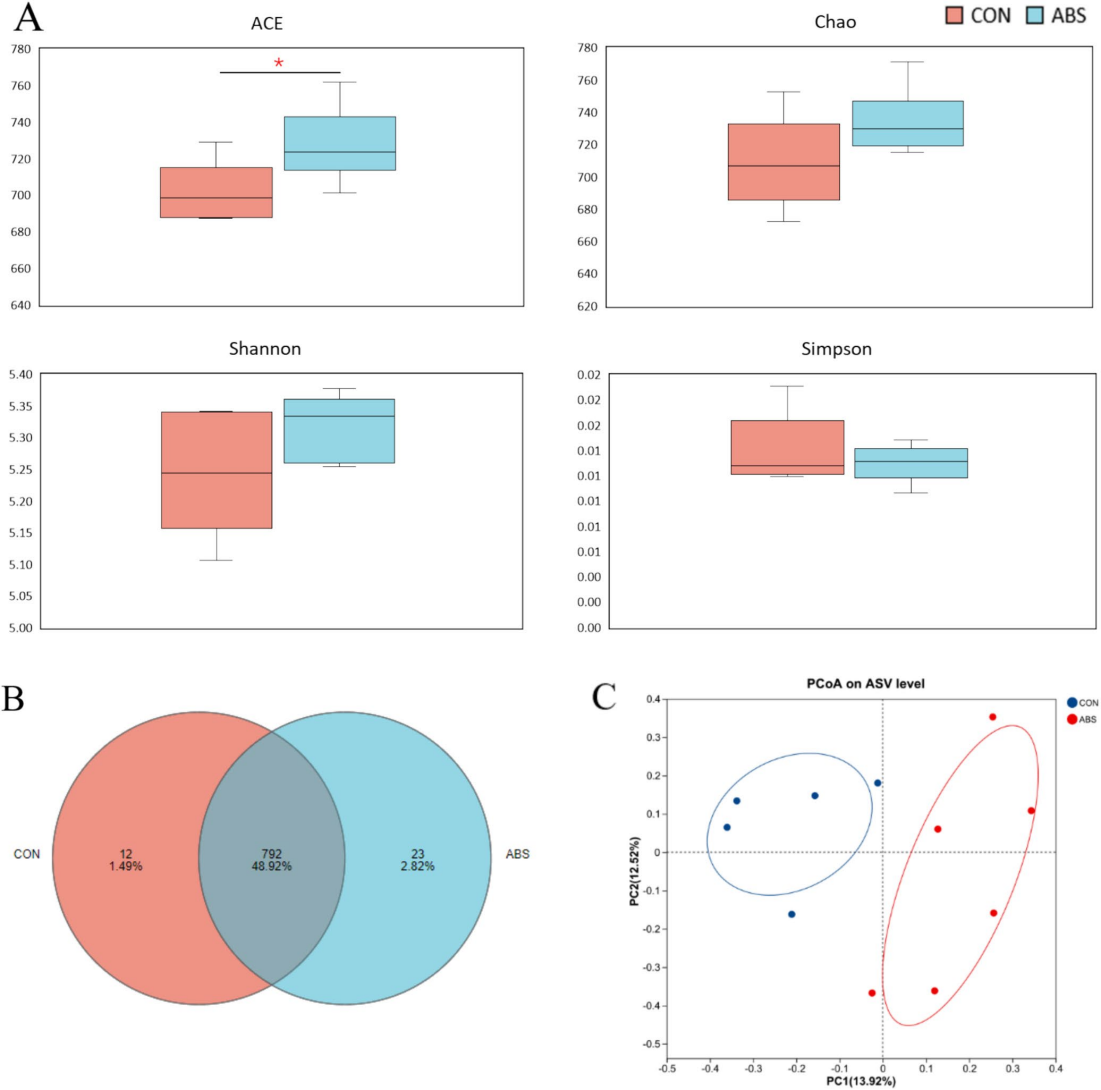
Effects of ABS on the rumen bacterial community richness and diversity in dairy heifers. **(A)** Alpha-diversity indexes of the rumen bacteria. **(B)** Venn diagram of the rumen bacterial community. **(C)** Principal coordinate analysis (PCoA) plot of the rumen bacterial community based on Bray–Curtis dissimilarity. *p < 0.05. CON, basal diet; ABS, basal diet including 150 g·DM/heifer daily of fresh ABS.

**Figure 2:**
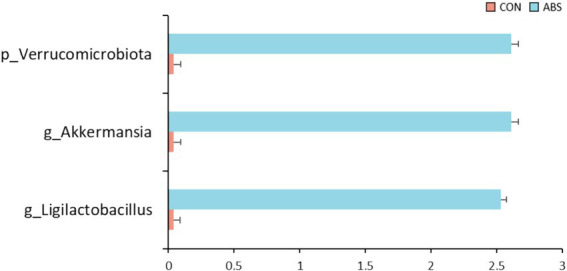
The differential bacteria at the phylum and genus levels in the rumen digesta between the CON and ABS groups. CON, basal diet; ABS, basal diet including 150 g·DM/heifer daily of fresh ABS.

The effects of ABS on the *α* and *β* diversity of the rumen bacterial community are presented in Figure 1 and Supplementary Table S1. Supplementation of ABS significantly increased the community richness including Sobs and ACE indices of the ASV level compared with the control group (*p* ≤ 0.05), but did not affect the Chao, Shannon, and Simpson indices (*p* > 0.05) (Figure 1A and Supplementary Table S1). The Venn diagram revealed that two groups shared 792 common ASVs, while 12 and 23 unique ASVs were illustrated in CON and ABS groups, respectively (Figure 1B). PCoA based on the Bray-Curtis distance showed clear separation in rumen microbial structure between the CON and ABS groups (Figure 1C). The effects of ABS on the relative abundances of ruminal bacteria phyla and genera are presented in Figure 2 and Supplementary Table S2. In the rumen, the dominant phyla for both groups were Bacillota (Firmicutes, 49.08%) and Bacteroidota (Bacteroidetes, 44.98%), with the ratio of Bacillota/Bacteroidota were 1.03 and 1.25 in CON and ABS groups, respectively. At the phylum level, heifers fed ABS had a higher relative abundance of Verrucomicrobiota (*p* ≤ 0.05). At the genus level, supplementation of ABS increased (*p* ≤ 0.05) the relative abundance of the ruminal bacteria genera *Akkermansia* (member of the Bacillota phylum) and *Ligilactobacillus* (member of the Bacillota phylum) in the ABS group compared with the CON group.

### 3.5 Correlations between rumen microbiota and physiological parameters

**Figure 3:**
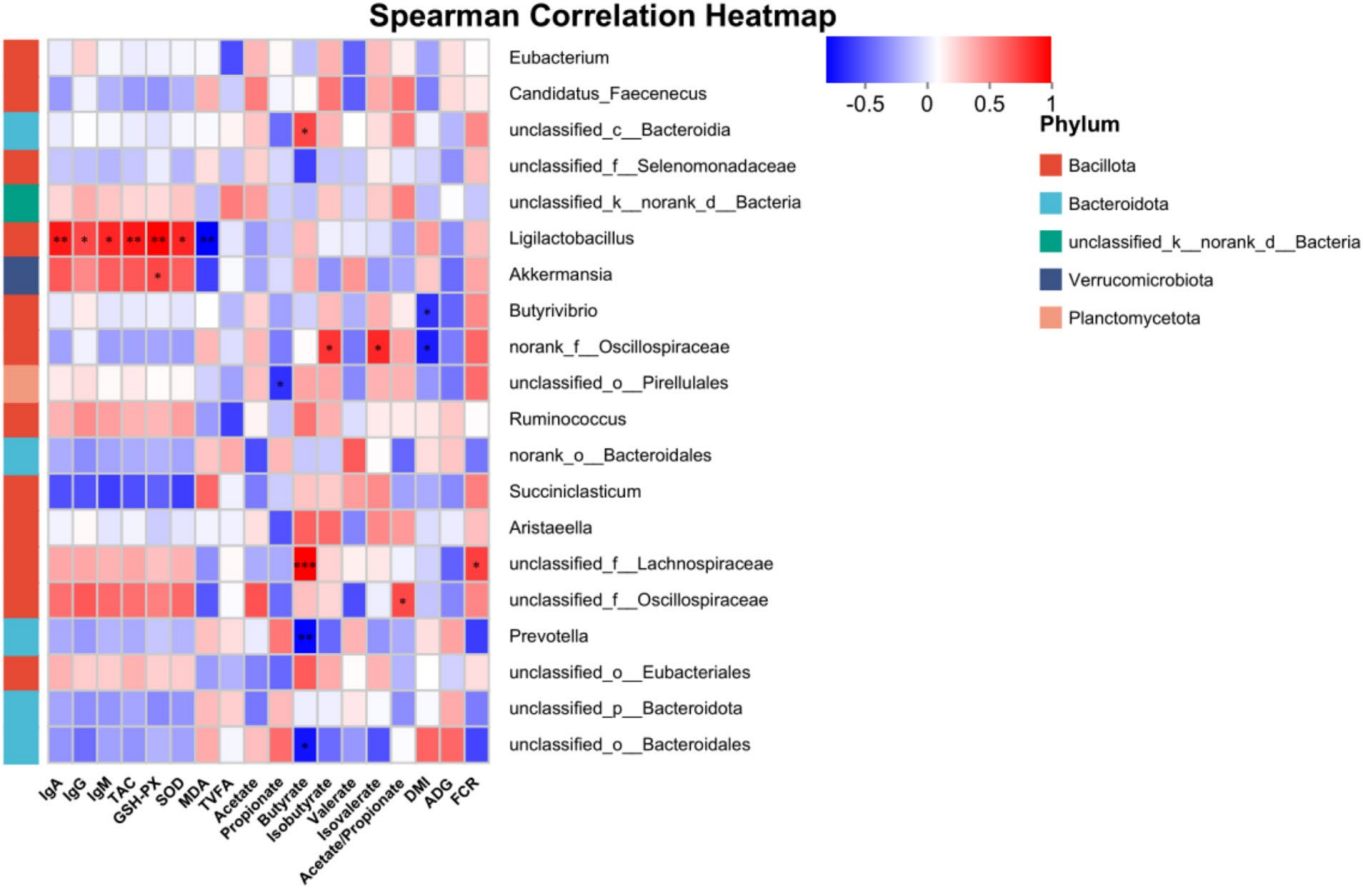
Correlation analysis of rumen microbiota and physiological parameters in dairy heifers. Red indicates a positive correlation, blue indicates a negative correlation. *p < 0.05, **p < 0.01, ***p < 0.001. IgA, immunoglobulin A; IgG, immunoglobulin G; IgM, immunoglobulin M; TAC, total antioxidant capacity; GSH-Px, glutathione peroxidase; SOD, superoxide dismutase; MDA, malondialdehyde; TVFA, total volatile fatty acids; DMI, dry matter intake; ADG, average daily gain; FCR, feed conversion ratio.

The correlations between the top 20 bacterial genera and physiological parameters were analyzed using Spearman correlation analysis (Figure 3). *Ligilactobacillus* was significantly positively correlated with IgA, IgG, IgM, TAC, GSH-PX, and SOD, but significantly negatively correlated with MDA (*p* ≤ 0.05). A highly significant positive correlation existed between *Akkermansia* and GSH-PX, and a highly significant negative correlation existed between *Butyrivibrio* and DMI (*p* ≤ 0.05). The molar proportion of butyrate was positively correlated with *unclassified_c__Bacteroidia* and *unclassified_f__Lachnospiraceae*, while negatively correlated with *Prevotella* and *unclassified_o__Bacteroidales* (*p* ≤ 0.05). *Norank_f__Oscillospiraceae* exhibited positive correlations with the molar proportion of isobutyrate and isovalerate whereas it was negatively correlated with DMI (*p* ≤ 0.05).

## 4 Discussion

In the present study, we compared the ADG, feed conversion ratio, as well as feed digestibility and utilization, blood immunity and antioxidant capacity, rumen fermentation patterns and microbial abundance. The main results of this study suggest that there is a potential to improve the growth performance of heifers by the addition of ABS to the diet.

Our results show that the heifers in the two groups had similar DMI despite the supplementation of ABS. However, the heifers fed the control diet faced challenges, as evidenced by a significantly lower ADG compared with heifers fed the diet supplementation with ABS. This variation may be attributable to the polysaccharides in the ABS cell wall, which have a growth-promoting effect on animals (35). Not surprisingly, ABS has a tendency to improve the feed conversion ratio of heifers. Similar to our results, Dong et al. (11) indicated that whereas the body weight of broilers did not respond to the addition of ABS (20 g/kg·DM) in diet, dietary ABS reduced feed intake and thus increased the feed conversion ratio of broilers. Numerous studies have shown that ABS contains rich nutrients such as protein, healthy substances such as antioxidants, and important bioactive substances such as phenols, which comprehensively support the beneficial effects on the growth performance of heifers [see, e.g., review by Yang et al. (9)]. In the previous study, however, there was no effect on the growth performance of the lambs and only a reduction in the feed cost when ABS was added to the diet (18). The inconsistency with the results of this study may be attributed to the proportion of ABS added to the lamb diet reached 10 and 20% respectively, and the ME of ABS was 20.6% lower than that of dietary alfalfa, which may affect the initial energy and nitrogen balance of the diet.

Research in human nutrition and medicine has shown that *Agaricus bisporus* has antioxidant and immune-boosting properties (8), and is considered a natural prebiotic (36). In the current study, heifers fed ABS had on average 10.4, 22.01, and 17.7% greater IgG, IgA, and IgM than that offered the control diet, respectively. Dietary *Agaricus bisporus* stimulates salivary secretory immunoglobulin A secretion in humans, which is known to be the first line of adaptive humoral immune defense of mucosal surfaces. A large number of studies have shown that *Agaricus bisporus* polysaccharides have the biological activity of immunomodulatory (37–39), which contributed to an increase in the level of immunity in heifers. In the present study, the addition of ABS resulted in lower blood serum levels of MDA, indicating weakened peroxidation. A decrease in MDA levels was accompanied by an increase in SOD, GSH-Px, and TAC, which was important for the activation‌‌ of oxygen free radicals. On the other hand, several studies have shown that *Agaricus bisporus* is a food source of antioxidants (6), such as *Agaricus bisporus* polysaccharides, which have biological activities in antioxidant (40). In agreement with our findings, Yang et al. (9) demonstrated that ABS as a dietary additive increased SOD activity and GSH-Px activity in serum, and also increased SOD activity but decreased MDA content in the yolk of laying hens. These help to explain the results observed in this trial, where ABS improved the serum immunity and antioxidant indicators in heifers.

In the nylon bag and gas production techniques test, *Agaricus bisporus* had slow rumen degradability and fermentability and did not affect total VFA concentration (10). In another previous study, *Agaricus bisporus* had no effect on rumen microbial protein concentrations in sheep (42). Consistent with these findings, there was no change in rumen fermentation parameters of heifers fed the ABS diets in this study. The promotion of feed digestion by adding mushrooms has previously been reviewed (8, 9). In the review by Mohan et al. (17), mushroom products such as mycelia, stems, dried powder, and extracts have been widely used in aquaculture, which can improve the digestive enzyme activity of the gastrointestinal tract of cultivable aquatic animals. More recently, zebrafish fed a synbiotic combination (*Agaricus bisporus* and probiotics) showed higher digestive enzyme activity (43). Moradzadeh-Somarin et al. (42) reported that replacing alfalfa with *Agaricus bisporus* to 210 g/kg·DM in sheep diets increased OM digestibility and metabolizable energy, but decreased NDF and CP digestibility. Nevertheless, we observed that adding ABS to the diet increased NDF digestibility by 4.65% and ADF digestibility by 4.56% in heifers. This variation may be attributed to differences in the inherent physicochemical composition of *Agaricus bisporus* and alfalfa, as well as the different species of experimental animals (44).

Regarding rumen microbiota, although ABS has been recognized as an additive or a component of animal feed (10, 41), its effect on rumen microbiota community structure is still unclear. Bacillota and Bacteroidota are two key bacteria in the rumen, and the Bacillota/Bacteroidota ratio is usually positively correlated with feed efficiency and milk yield of dairy cows (45). The Bacillota/Bacteroidota ratio of the ABS group was higher than that of the CON group (1.25 vs. 1.03), which supported the higher growth performance of the ABS group in this study. At the phylum level, ABS only increased the relative abundance of Verrucomicrobiota in heifers in this experiment. Verrucomicrobota is considered a major degrader of glycosaminoglycans due to its presence of glycoside hydrolases and sulfatases (46). Therefore, Verrucomicrobota contains species that are highly specialized in degrading complex polysaccharides (47). A disproportionately high number of carbohydrate-active enzymes per Mb of the genome suggests that Verrucomicrobiota has a significant metabolic capacity to support rumen function, even though it is not very abundant in the rumen, according to research by Gharechahi et al. (48). A recent study reported that the Verrucomicrobiota genome has the second-highest metabolic capacity for carbohydrate polysaccharides, trailing only the Bacteroidota genome among rumen bacteria (48). Further analysis of the taxonomic origin of enzymes using secretion signals revealed that 36% of the enzymes secreted by Verrucomicrobiota act on the degradation of rumen lignocellulose (48). Liu et al. (49) demonstrated that the abundance of Verrucomicrobiota in the rumen of cattle in the high-cellulose forage group was higher than that in the low-cellulose forage group. The majority of the Verrucomicrobiota in the rumen of Hu sheep belong to the genus *Akkermansia* (50), which is enriched in the gastrointestinal tract of animals fed crude fiber diet. In the present study, *Akkermansia* was enriched in the rumen of heifers fed the ABS diet. Similarly, *Agaricus bisporus* polysaccharides increased the relative abundance of *Akkermansia* in the gastrointestinal tract of mice (49). These findings support the results that ABS improved the digestibility of NDF and ADF in heifers in this trial. In addition, Fujio-Vejar et al. (51) found that Verrucomicrobia has anti-inflammatory properties. Remarkably, *Akkermansia muciniphila* was the first cultured representative of Verrucomicrobia at the species level, and there was a positive correlation between host immunity and *Akkermansia muciniphila* abundance (52). The rumen of heifers supplemented with ABS had a higher abundance of *Akkermansia muciniphila* in this experiment (Supplementary Table S2). These may explain why dietary ABS improved the serum immune and antioxidant indexes of heifers. *Ligilactobacillus* is made up of former members of *Lactobacillus* and is a type of lactic acid bacterium that benefits gastrointestinal health (53). Moreover, Li et al. (54) found that *Ligilactobacillus* was positively correlated with intestinal anti-inflammatory. Our results also showed that *Ligilactobacillus* was positively correlated with serum immune and antioxidant parameters. The genus *Ligilactobacillus* and species *Ligilactobacillus murinus* were abundant in the rumen of heifers in the ABS group (Supplementary Table S2), suggesting again that ABS was beneficial to the growth and immunity of heifers.

## 5 Conclusion

The findings observed in this study suggested that ABS supplementation improves growth performance by increasing ADG and promoting the feed conversion ratio of dairy heifers. Additionally, our results suggest ABS has the ability to both enhance the immunity and antioxidant capacity of heifers. The ABS was effective in nutrient digestion because NDF and ADF digestibilities were greater in ABS heifers. Feeding ABS enhanced some ruminal bacteria populations, including Verrucomicrobia, *Akkermansia*, *Akkermansia muciniphila*, *Ligilactobacillus,* and *Ligilactobacillus murinus*, which may partially explain the higher nutrient digestibility, blood indicators, and growth performance in ABS heifers.

## Data Availability

The raw data reads of 16S rRNA gene sequencing of microbiota are deposited in the National Center for Biotechnology Information (NCBI) repository, accession number PRJNA1224486.

## Footnotes

^1^ http://www.mushroommarket.net
