# Supplementary material for: Agaricus bisporus stipe fed to dairy heifers: effects on growth performance, immunity and antioxidant capacity, and rumen microbiota
Source: Front Vet Sci. 2025 Mar 21;12:1556330. doi: 10.3389/fvets.2025.1556330 (PMC11969463; doi:10.3389/fvets.2025.1556330)
Supplement: Supplementary file 1 [file Table_1.DOCX]

**Supplementary Information**

TABLE S1 Effects of ABS on the rumen bacterial alpha diversity indices in dairy heifers.

| Item | Treatment^1^ | | SEM | *P*-value |
| --- | --- | --- | --- | --- |
|  | CON | ABS |  |  |
| Sobs | 632.8 | 655.3 | 5.852 | 0.048 |
| ACE | 700.9 | 727.4 | 6.840 | 0.046 |
| Chao | 708.8 | 734.1 | 8.062 | 0.122 |
| Shannon | 5.25 | 5.32 | 0.025 | 0.155 |
| Simpson | 0.01 | 0.01 | 0.001 | 0.488 |

^1^CON = basal diet; ABS = basal diet including 150 g∙DM/heifer daily of fresh ABS.

TABLE S2 Effects of ABS on the rumen bacterial community at the phylum and genus levels in dairy heifers.

| Item | Treatment^1^ | | SEM | *P*-value |
| --- | --- | --- | --- | --- |
|  | CON | ABS |  |  |
| Phylum level |  |  |  |  |
| Bacteroidota | 48.29 | 41.66 | 2.243 | 0.149 |
| Bacillota | 47.35 | 50.80 | 1.919 | 0.398 |
| Planctomycetota | 1.94 | 1.73 | 0.327 | 0.768 |
| Verrucomicrobiota | 0.04 | 2.61 | 0.548 | 0.009 |
| unclassified_k__norank_d__Bacteria | 1.10 | 1.05 | 0.123 | 0.857 |
| Pseudomonadota | 0.68 | 1.43 | 0.228 | 0.099 |
| Genus level |  |  |  |  |
| *unclassified_o__Bacteroidales* | 23.18 | 20.76 | 1.230 | 0.353 |
| *unclassified_p__Bacteroidota* | 9.47 | 7.70 | 0.604 | 0.152 |
| *unclassified_o__Eubacteriales* | 8.03 | 8.79 | 0.479 | 0.462 |
| *Prevotella* | 8.84 | 6.51 | 1.350 | 0.419 |
| *unclassified_f__Oscillospiraceae* | 6.62 | 6.94 | 0.336 | 0.658 |
| *unclassified_f__Lachnospiraceae* | 5.83 | 7.50 | 0.591 | 0.173 |
| *Aristaeella* | 6.09 | 5.05 | 0.656 | 0.462 |
| *Succiniclasticum* | 5.37 | 5.39 | 0.450 | 0.984 |
| *norank_o__Bacteroidales* | 5.05 | 4.85 | 0.661 | 0.888 |
| *Ruminococcus* | 4.26 | 4.42 | 0.604 | 0.904 |
| *unclassified_o__Pirellulales* | 1.65 | 1.20 | 0.328 | 0.530 |
| *norank_f__Oscillospiraceae* | 1.55 | 1.26 | 0.161 | 0.405 |
| *Butyrivibrio* | 1.50 | 1.04 | 0.259 | 0.400 |
| *Akkermansia* | 0.04 | 2.61 | 0.548 | 0.009 |
| *Ligilactobacillus* | 0.04 | 2.53 | 0.481 | 0.002 |
| *unclassified_k__norank_d__Bacteria* | 1.10 | 1.05 | 0.123 | 0.857 |
| Specie level |  |  |  |  |
| *unclassified_o__Bacteroidales* | 23.18 | 20.76 | 1.230 | 0.353 |
| *unclassified_p__Bacteroidota* | 9.47 | 7.70 | 0.604 | 0.152 |
| *unclassified_o__Eubacteriales* | 8.03 | 8.79 | 0.479 | 0.462 |
| *unclassified_f__Oscillospiraceae* | 6.62 | 6.94 | 0.336 | 0.658 |
| *unclassified_f__Lachnospiraceae* | 5.83 | 7.50 | 0.591 | 0.173 |
| *Succiniclasticum_ruminis* | 5.37 | 5.39 | 0.450 | 0.984 |
| *unclassified_g__Prevotella* | 4.47 | 4.02 | 0.465 | 0.659 |
| *Bacteroidales_bacterium_CF* | 3.79 | 3.50 | 0.481 | 0.784 |
| *unclassified_g__Aristaeella* | 3.80 | 3.12 | 0.404 | 0.429 |
| *Prevotella_ruminicola* | 3.99 | 2.28 | 0.936 | 0.391 |
| *Ruminococcus_bromii* | 2.98 | 2.98 | 0.524 | 1.000 |
| *Aristaeella_hokkaidonensis* | 2.28 | 1.93 | 0.262 | 0.533 |
| *unclassified_o__Pirellulales* | 1.65 | 1.20 | 0.328 | 0.530 |
| *Ruminococcaceae_bacterium_AB4001* | 1.47 | 1.12 | 0.164 | 0.320 |
| *rumen_bacterium_YS1* | 1.26 | 1.35 | 0.343 | 0.911 |
| *Akkermansia_muciniphila* | 0.04 | 2.61 | 0.548 | 0.009 |
| *Ligilactobacillus_murinus* | 0.04 | 2.53 | 0.481 | 0.002 |
| *unclassified_k__norank_d__Bacteria* | 1.10 | 1.05 | 0.123 | 0.857 |
| *unclassified_g__Ruminococcus* | 0.93 | 1.16 | 0.164 | 0.516 |

^1^CON = basal diet; ABS = basal diet including 150 g∙DM/heifer daily of fresh ABS

Only bacteria with a relative abundance greater than 1% in the rumen of at least one group of heifers were shown.
